# Does feeling what you eat change how you eat? Implications of an intervention to promote consciousness of eating experiences

**DOI:** 10.3389/fpsyg.2023.1229105

**Published:** 2024-01-04

**Authors:** Carina Carlucci Palazzo, Barbara Esteves Leghi, Rosa Wanda Diez-Garcia

**Affiliations:** ^1^Internal Medicine Department, Ribeirão Preto Medical School, University of São Paulo (USP), Ribeirão Preto, Brazil; ^2^Laboratory of Food Practices and Behaviour – PrátiCA, University of São Paulo (USP), Ribeirão Preto, Brazil; ^3^Department of Health Sciences, Ribeirão Preto Medical School, University of São Paulo (USP), Ribeirão Preto, Brazil

**Keywords:** nutritional trial, mixed methods, consciousness, eating behavior, food and nutrition education

## Abstract

**Introduction:**

This work aims to understand the experience of participating in the Food and Nutrition Education Program workshops with Sensory and Cognitive Exercises (PESC) and measure its impact on eating behavior.

**Methods:**

The PESC consists of four workshops with exercises stimulating perception and reflection on bodily sensations triggered in eating situations. It was developed to promote consciousness of eating experiences in women who reported difficulty in controlling their body weight and who increased over 5% of their body weight in the previous year. This is a mixed-methods study designed as a controlled trial. The intervention group (*n* = 19) was evaluated before the first workshop (T0) and after the last workshop (T1) and the control group (*n* = 18), which did not participate in the workshops, was evaluated twice (T0 and T1), with an interval of 3 to 4 weeks. At T0 and T1, it was applied the Intuitive Eating Scale (IES-2) and the Three Factor Eating Questionnaire (TFEQ-R21). In T1, the intervention group also participated in a qualitative interview.

**Results:**

After participating in the PESC, the intervention group showed an increase in the IES-2 total score (95% Confidence Interval = (0.10, 0.39)) and a decrease in the emotional eating scale score (95% Confidence Interval = (−16.03; −3.85)). The interviews’ analysis revealed the participants’ self-observation process, which led to the perception of their practices, priorities, intentions, eating difficulties, and the connection between these aspects and environmental issues. The following themes were considered: Food management/control, Body dissatisfaction, Reflection/re-signification, and Different ways of being in familiar contexts.

**Discussion:**

The results allow us to infer that, after participating in the PESC, the intention to manage food intake became more effective, less susceptible to oscillations imposed by the external environment, and more supported by internal resources.

## 1 Introduction

Promoting perception, recognition, and appreciation of body sensations triggered by environmental and physiological stimuli has been identified as an essential path in the search for new approaches to improve dietary patterns and manage obesity (Shepherd, 2012; Simmons and DeVille, 2017; Herbert, 2020). The perception of body sensations is fundamental to recognizing the body as a subject of life experiences (Herbert and Pollatos, 2012) and regulating adaptive behavior (Herbert, 2020). In food, it is known that eating disorders and obesity are associated with changes in the perception, recognition, and appreciation of body sensations indicative of physiological states linked to homeostasis (Simmons and DeVille, 2017).

In this context, and considering the existing demand for the development of new approaches aimed at improving dietary patterns (Clifford et al., 2015), the Food and Nutrition Education Program with Sensory and Cognitive Exercises (PESC) was developed, having as its central axis the promotion of consciousness of eating experiences in women with difficulty maintaining body weight. The PESC comprises exercises that stimulate perception and reflection about the bodily sensations triggered in situations associated with the current food scenario (Palazzo et al., 2021).

In works already published, it was demonstrated that the PESC was able to increase interoceptive sensitivity (sensitivity to bodily physiological sensations), to promote a greater expression of exteroceptive perception (perception and processing of environmental sensory stimuli) (Palazzo et al., 2022), and to promote reflection on the embodied eating experiences incited by the intervention (Leghi et al., 2022). In this way, the PESC represents an essential step in the search for dietary interventions capable of promoting the perception of bodily sensations triggered by environmental and physiological stimuli.

For the development of the PESC, the theory of consciousness proposed by Pereira-Junior was considered (Pereira Jr, 2013), according to which cognitive contents become conscious when associated with a sense/meaning, that is, when an environmental stimulus is recognized as a lived experience. An important aspect of conscious experiences is modifying how individuals perceive and react to the environment, generating new adaptive behaviors in face of environmental stimuli (Pereira Jr, 2014).

It was also considered the importance of the so-called obesogenic environment as a promoter of opportunities and conditions that favor the excessive consumption of food, predominantly industrialized and hyper-palatable foods (Lake, 2018), in an intuitive and unreflected way (Wansink, 2010). The perception, recognition, and reflection about environmental stimuli to food consumption associated with the perception, recognition, and appreciation of body cues can make individuals less vulnerable to environmental stimuli that lead to excessive eating (Simmons and DeVille, 2017).

PESC has similarities with other programs that promote perception and appreciation of body signs, such as Intuitive Eating (Tribole and Resch, 2012), or that stimulate attention and perception of experiences such as mindfulness-based interventions (Kristeller and Wolever, 2011; Timmerman and Brown, 2012). On the other hand, unlike other approaches, the PESC is grounded in a theory of consciousness (Pereira Jr, 2013, 2014). Furthermore, it brings innovations by jointly proposing the perception, reflection, and meaning of environmental stimuli associated with the current food scenario, with the perception and appreciation of body signals triggered by these environmental stimuli. In other words, the PESC aims to promote consciousness of how environmental stimuli affect eating experiences and lead to undesirable eating patterns.

This work seeks to evaluate the PESC measuring its impact on aspects of eating behavior and understanding the experience of participating in the workshops of PESC from the participants’ perspective.

## 2 Methods

Ethical approval for this study was obtained by the Research Ethics Committee of the Clinical Hospital of Ribeirão Preto (HCRP-USP), protocol 3.335.083. All participants in this study signed the free and informed consent form.

### 2.1 Study design

This is a parallel convergent mixed methods study. This means that quantitative and qualitative data were collected and analyzed separately, to then go through an additional and integrative analysis step (Creswell and Clark, 2013).

The application and assessment of this intervention should, traditionally, be done through a controlled trial (Victora et al., 2004). On the other hand, the complexity of the topic addressed and the intention to explore how the intervention group participants perceive and appropriate the content presented during the intervention in their particular contexts required the use of qualitative instruments (Marshall and Rossman, 2016).

The study design is characterized as a controlled trial (Chan et al., 2013) with an assessment of the intervention group (I-PESC) before (T0) and after (T1) participation in the PESC and an assessment of the control group (C-PESC) in two moments (T0 and T1), with a 3 to 4-week interval between each assessment (without participation in the intervention).

Qualitative methods in intervention studies and clinical trials, especially in complex interventions (Craig et al., 2008) such as the one in this study, have been advocated for some years (Sandelowski, 1996). Particularly in health education, the use of mixed methods is carried out to add depth and meaning to the empirical findings (Zhang, 2014), as well as favoring the understanding of the effects of the intervention in specific contexts (Johnson and Schoonenboom, 2016).

### 2.2 Intervention

The PESC was developed to promote consciousness of eating experiences. The PESC consisted of four weekly workshops lasting 2 h and three exercises performed between each workshop.

In the workshops, activities were applied based on everyday situations, which call attention and promote reflection on the connection between environmental stimuli, body sensations, and eating outcomes. In each workshop, sensory and cognitive aspects of eating experiences were explored based on the topics: (1) the senses and the desire to eat; (2) the senses and food pleasure; (3) hunger and satiety: how we deal with bodily cues; and (4) how we record experiences in the body.

The proposed exercises are followed by reflection and cognitive connection with everyday experiences previously lived by the participants to promote consciousness of eating experiences (Pereira Jr, 2013, 2014).

The PESC application protocol, including a detailed description of the activities, was previously published (Palazzo et al., 2021). All workshops were conducted by the main and second researcher of this study, accompanied and supervised by the third researcher of the project.

### 2.3 Participants

The call for participation in the research were disseminated through social networks, the university’s e-mail, and published on the university’s website. The screening process involved applying the inclusion and exclusion criteria and assessing the availability of time to participate in the workshops. The recruitment took place during the coronavirus pandemic, so the participants selection for I-PESC (whose participation in the research would occur in groups) and for C-PESC (whose participation in the research would occur individually) was made according to the possibility of conducting face-to-face meetings in each condition. The recruitment for participation in I-PESC and C-PESC involved two different consent forms, since the procedures for each group were different. The participants did not know about the existence of two study groups.

Inclusion criteria were adult women (aged between 20 and 59 years old) with BMI between 18.5 and 34.9 kg/m2, self-reported difficulty maintaining body weight (with weight gain greater than 5% of body weight in the last 12 months) and a desire to improve their relationship with food. Women using a psychotropic medication, smokers, and women with a BMI equal to or greater than 35 kg/m2 were excluded due to the change in taste caused by these conditions (Turjanski and Lloyd, 2005; Pepino and Mennella, 2007; Kure Liu et al., 2019). Nutritionists and nutrition students were also considered exclusion criteria, which could constitute a bias in this study. Pregnant and lactating women were excluded due to these conditions’ dietary and behavioral specificities. Finally, women with allergies or intolerance to any foods used in the intervention were also excluded.

Between September 2019 and December 2020, fifty-four women were selected and allocated to the I-PESC and C-PESC groups. The I-PESC group consisted of 36 participants, of which 19 completed all stages of the intervention. All 18 participants allocated to the C-PESC completed the study stages. Dropouts occurred due to a lack of time to participate in all workshops.

### 2.4 Anthropometric and sociodemographic characterization

The body weight (Kg) and height (m) of the participants was measured. A standard protocol was applied to all procedures (Lipschitz, 1994). Information was collected on weight variation in the last 12 months, such as age, marital status, education, and *per capita* income.

### 2.5 Questionnaires

Eating behavior was assessed using two questionnaires at T0 and T1 in the I-PESC and C-PESC groups.

The Intuitive Eating Scale (IES-2) is a 23-item self-administered questionnaire that assesses an individual’s tendency to trust and follow their body cues of hunger and satiety to the detriment of external stimuli to food consumption. To this end, it considers four subscales: unconditional permission to eat (UPE), eating for physical rather than emotional reasons (EPRER), reliance on hunger and satiety cues (RHSC), and body-food choice congruence (BFCC). In this study, the version translated into Portuguese was used, in which each item of the IES-2 is scored on a five-point scale ranging from 1 (never) to 5 (always). Higher total scores indicate greater confidence in physiological hunger and satiety (da Silva et al., 2018). The IES-2 is recommended for assessing nutritional approaches since it is positively related to self-esteem and satisfaction with life and negatively related to eating disorder symptoms and body mass index (BMI) (Tylka and Kroon Van Diest, 2013).

The Three-Factor Eating Questionnaire (TFEQ – R21) is a 21-item self-administered questionnaire consisting of 3 scales: cognitive restriction, emotional eating, and uncontrolled eating (Natacci and Ferreira Junior, 2011). In this study, the version translated into Portuguese was used, in which the cognitive restriction scale is composed of 6 items and measures the self-imposition of dietary control to maintain or lose body weight; the emotional eating scale consists of 6 items and assesses the propensity for loss of food control and overconsumption in response to negative emotional states, such as anxiety, loneliness, and depression; the uncontrolled eating scale consists of 9 items, and verifies the tendency to lose eating control due to hunger or environmental stimuli. Each item must be answered on a 4-point scale, except the last item on the cognitive restriction scale, which has 8 points. The average of each of the scales is calculated and transformed into a scale from 0 to 100 points (Natacci and Ferreira Junior, 2011). The TFEQ-R21 has been widely applied in studies on eating behavior (Ferreira et al., 2019; Szakály et al., 2020; Lin et al., 2021; Guivarch et al., 2022) since its scales are considered good predictors of food vulnerability in obesogenic environments (Bryant et al., 2019).

### 2.6 Interview

Qualitative interviews, as a research tool, aim to build knowledge based on reflection and interaction between the researcher and the participants in the intervention and are characterized by the simultaneous search for factual aspects and the meaning of the lived experiences (Kvale and Brinkmann, 2009).

In this study, the interviews were applied only to the I-PESC group at T1 and were conducted using a script of questions. The interviews aim to bring particular and contextual elements to understanding the intervention’s participation process.

The interview proposes an initial reflection on the participant’s relationship with food prior to participating in the PESC, and then narrows down to aspects relevant to the intervention. The participants’ perception of the content worked on in the workshops was explored, and how these are connected to the conceptions about food already brought by the participants, as well as possible repercussions of the activities of the workshops on the eating experiences throughout the intervention period.

The script used considered the following questions:

1. Talk about what your relationship with food is like.
2. What was the role of the workshops for you during this period? What makes you say that?
3. What were your major discoveries after the workshops?
4. During these weeks, have you noticed any changes in your diet?
5. Based on this experience, what do you think you need to invest in your relationship with food?

The interviews were recorded for later transcription and analysis of the material.

### 2.7 Data analysis

#### 2.7.1 Statistical analysis

All variables were subjected to descriptive analysis.

The sample characterization variables were analyzed using Student’s t-test for independent samples and considering a 95% confidence interval.

A linear regression model with mixed effects was used to compare the I-PESC and C-PESC groups at T0 and T1 and to evaluate the variation between T0 and T1 in I-PESC or C-PESC. A 95% confidence interval was considered. Confidence intervals that do not include the zero value show evidence of a statistical difference. In contrast, their limits indicate the magnitude of that difference.

All statistical analyses were performed using Statistical Analysis Software (version 9.3, SAS Institute, Inc., Cary, NC, United States).

It was the hypothesis of this study that, after participating in the PESC, the participants would increase their scores on the IES-2 and decrease their scores on the TFEQ-R21 subscales.

#### 2.7.2 Interview analysis

The interviews were transcribed in full and analyzed using the ATLAS.ti® software (version 9.0, GmbH, Berlin, Germany). The Reflective Thematic Analysis was carried out as described by Braun & Clarke (Braun and Clarke, 2006, 2019), using the theory of consciousness to develop the PESC (Pereira Jr, 2013, 2014) as a theoretical framework. The leading researcher did the reading and the first codification of the material. Then they discussed with the study supervisor the final version of topics and sub-topics that gave rise to the interpretative map, configured as a visual support for understanding how the proposed topics and sub-topics are related (Braun and Clarke, 2006).

## 3 Results

### 3.1 Sample characterization

The participants in this study were primarily single (*n* = 18, 49%), and had completed higher education (*n* = 29, 78%). Table 1 shows the other characteristics evaluated in the sample characterization. The intervention and control groups did not differ in any considered aspects.

**Table 1:** Sample characterization.

	I-PESC (*n* = 19)	C-PESC (*n* = 18)	*p*	95% confidence interval (Groups difference)
Age (years)	36.78 ± 12.73	36.00 ± 12.53	0.850	−7.6; 9.2
Income (R$)	3752.78 ± 3012.54	4117.65 ± 2232.78	0.685	−2185.4; 1455.7
Body weight (Kg)	73.31 ± 9.08	75.26 ± 13.98	0.620	−9.9; 6.0
BMI (Kg/m^2^)	28.18 ± 3.19	26.44 ± 4.42	0.181	−0.8; 4.3
Body weight variation (Kg)	5.36 ± 1.86	7.03 ± 3.65	0.096	−3.6; 0.3
Body weight variation (%)	8.05 ± 2.93	10.12 ± 4.83	0.128	−4.7; 0.6

I-PESC, intervention group; C-PESC, control group. R$, Brazilian reais. Results expressed as mean ± standard deviation.

### 3.2 Questionnaires

Table 2 shows the mean scores obtained on the Intuitive Eating Scale and the Three Fact Eating Questionnaire scales by the I-PESC and C-PESC groups at the evaluated moments. No differences were observed between I-PESC and C-PESC at T0.

**Table 2:** Eating behavior questionnaires scores at T0 and T1.

		T0	T1	Mean Difference	95% Confidence interval (CI)
IES-2 Total score	I-PESC	3.00 ± 0.53	3.25 ± 0.43	0.25	0.10; 0.39*
	C-PESC	3.28 ± 0.54	3.21 ± 0.59	−0.07	−0.20; 0.06
IES-2	I-PESC	3.54 ± 0.79	3.32 ± 0.83	−0.22	−0.46; 0.02
UPE	C-PESC	3.38 ± 0.74	3.60 ± 0.67	0.22	−0.02; 0.47
IES-2 EPRTER	I-PESC	2.64 ± 0.96	3.07 ± 0.84	0.42	0.21; 0.63*
	C-PESC	3.11 ± 0.82	3.02 ± 0.92	−0.09	−0.3; 0.12
IES-2 RHSC	I-PESC	2.84 ± 0.73	3.28 ± 0.78	0.43	0.10; 0.77*
	C-PESC	3.15 ± 0.76	3.24 ± 0.79	0.09	−0.25; 0.44
IES-2 BFCC	I-PESC	3.51 ± 0.81	3.79 ± 0.74	0.28	0.02; 0.54*
	C-PESC	3.74 ± 0.76	3.76 ± 0.57	0.02	−0.25; 0.29
TFEQ - UE score	I-PESC	48 ± 17	43 ± 13	−4.28	−9.95; 1.39
	C-PESC	42.39 ± 12.59	41.35 ± 17.29	−1.03	−7.92; 5.86
TFEQ - CR score	I-PESC	48 ± 17	55 ± 20	7.02	−0.28; 14.32
	C-PESC	51.54 ± 17.34	45.99 ± 17.02	−5.55	−12.75; 1.65
TFEQ - EE score	I-PESC	56 ± 20	46.20 ± 23.72	−9.94	−16.03; −3.85*
	C-PESC	49.07 ± 24.79	49.07 ± 24.49	−3.55	−10.14; 3.03

Results expressed as mean ± standard deviation. I-PESC, Intervention group. C-PESC, Control group. IES-2, Intuitive Eating Scale-2. UPE, Unconditional Permission to Eat subscale (IES-2). EPRTER, Eating for Physical Rather than Emotional Reasons subscale (IES-2). RHSC, Reliance on Hunger and Satiety Cues subscale (IES-2). BFCC, Body-Food Choice Congruence subscale (IES-2). TFEQ – UE, Uncontrolled Eating scale (Three Fact Eating Questionnaire). TFEQ – CR, Cognitive Restraint scale (Three Fact Eating Questionnaire). TFEQ – EE, Emotional Eating scale (Three Fact Eating Questionnaire). ^*^Indicates statistical difference between T0 and T1.

It can be observed that the intervention group showed an increase in the total score of the IES-2 due to the increase in the score in the EPRTER, RHSC, and BFCC subscales, which indicates a greater appreciation and confidence in the physiological cues of hunger and satiety as guides for consumption food after the intervention. In addition, there was a decrease in the emotional eating scale score after participation in the PESC, which indicates a reduction in emotional eating in response to negative emotions.

### 3.3 Interviews

The interviews lasted an average of 9 min, ranging from 5 to 12 min. Table 3 presents the themes and sub-themes constructed from the analysis of the interviews accompanied by illustrative quotes. Quotes presented throughout the results section use pseudonyms to protect the identity of participants.

**Table 3:** Themes, sub-themes, and illustrative quotes of the interviews.

Themes	Sub-themes	Quote
FOOD MANAGEMENT/CONTROL	–	*“For example, I never eat two loaves of bread; I eat a bun for breakfast, that delicious bread that I make, because I want to taste it, I want to feel the pleasure of eating something I really like. But I never eat another; Because I am disciplined.” (Camila)*
BODY DISSATISFACTION	–	*“When I got married, I weighed 48 kilos (laughs). Wow, I was always skinny as a child and young woman. That changed when I tried to get pregnant, took the hormones, and things got a little messed up. So now I try to control myself.” (Ema)*
REFLECTION / RE-SIGNIFICATION	–	*“… one thing I noticed a lot in the last few weeks was the satiety issue. I do not know, I did not know – to be more optimistic – I do not know when I’m full. So now I can stop, I ask, do I want to eat this? Am I already full, or is it just to not leave it on the plate? And it was a cultural thing built by my family and school… so now I can question it.” (Wanda)*
DIFFERENT WAYS OF BEING IN FAMILIAR CONTEXTS	(1) Perception of own food desires. (2) Greater attention to body cues. (3) Search for new skills related to food.	*(1) “iFood is something I really want and will always be crap. So I already removed the notifications from my mobile, and now I do not even remember about it; I do not feel like it.” (Anna)* *(2) “… I paid attention to some things that sometimes we do not even think about… like this issue of feeling hungry and eating… it’s simple, right? It seems simple… eat when you feel hungry and stop eating when you are full. (…) Sometimes I’m eating, and I remember about it, and I think: I’m not hungry anymore; why am I continuing to eat?” (Bruna)* *(3) “I live alone, so what bothers me is that sometimes I buy that bunch of greens and vegetables, and they spoil! Then, wow, it leaves a bad taste to throw everything away. So I think it’s better to learn to freeze and do it in time; I think it’s less wasteful. And I think that’s something I still need to learn.” (Daniela)*

## 4 Discussion

### 4.1 Questionnaires

The increase in scores on the IES-2 scale and on the EPRTER, RHSC, and BFCC subscales, as well as the decrease in the emotional eating scale of the TFEQ-R21, indicate, respectively, a greater appreciation and confidence in the physiological cues of hunger and satiety as guides of the food consumption and a reduction in emotional eating in response to negative emotions, which is consistent with the expected outcome of the activities proposed by the PESC. It is known that interventions focused on attention and appreciation of body signals can result in an increase in the IES-2 score (Katcher et al., 2021) and improvements in eating behavior, including a decrease in emotional eating (Alberts et al., 2012; Warren et al., 2017; Schnepper et al., 2019), which, in general, is seen as cues of the potential of such interventions in the search for better eating patterns. Thus, this study’s results add to the literature’s findings.

Cross-sectional studies show that higher IES-2 scores are associated with fewer eating disorder symptoms (Tylka and Kroon Van Diest, 2013; Richard et al., 2019), lower BMI (Herbert et al., 2013; Tylka and Kroon Van Diest, 2013; Anderson et al., 2016), greater pleasure, and less food-related anxiety (Smith and Hawks, 2006). However, no intervention studies evaluated the effect of varying IES-2 scores in these parameters.

In a cross-sectional study carried out with adult Brazilian women, it was concluded that emotional eating, assessed by the TFEQ-R21, is positively related to BMI (Natacci and Ferreira Junior, 2011), corroborated by studies in other countries (Cornelis et al., 2014; O'Brien et al., 2016). A prospective study showed that after behavioral intervention for weight loss, participants who had a decrease in the emotional eating scale were also more successful in losing body weight in the 12 months following the intervention (Braden et al., 2016).

The results obtained through the questionnaires point to positive changes in the relationship with food, which may impact food content and/or situations involving food. However, the small sample size and short intervention period may have minimized the visibility of the impact of the PESC. Looking at the process of participation in the PESC through interviews can, on the other hand, provide a more in-depth and contextualized perception to complement the results discussed so far.

### 4.2 Interviews

The reports obtained in the interviews were marked by an intense exercise of self-observation, stimulated not only by the interview questions but also by a precise movement of externalization of a process triggered by the workshops. This self-observation resulted in the perception of their practices, priorities, intentions, and eating difficulties, and also in the connection between these aspects with environmental issues; that is, after the PESC, the participants were able to do a careful and reflective reading on the impact that elements of the environment exert on body sensations related to food and, as a consequence, on how eating experiences happen.

This contextualized and integrative self-observation, i.e., which considers how external stimuli impact emotions and food cravings and combine with other aspects such as beliefs, intentions, and physiological sensations, allows, at times, the expansion of this perspective, including new possibilities and attitudes towards food. Promoting this self-observation and reflection on different aspects involved in eating experiences (including environmental aspects and bodily sensations) is the central objective of the workshops, with the possibility of seeing different ways of being in contexts already known as one of the expected outcomes in conscious experiences (Pereira Jr, 2014). The narrative exercise promoted by the interviews is recognized as an essential tool for the perception and reflection on reality (Mollo et al., 2022) and, in this work, it contributed not only to its purpose of helping us to understand how the participants processed the activities of the workshops, but also it served as the final stage of this process of perception and development of consciousness of eating experiences promoted by the workshops.

**Figure 1:**
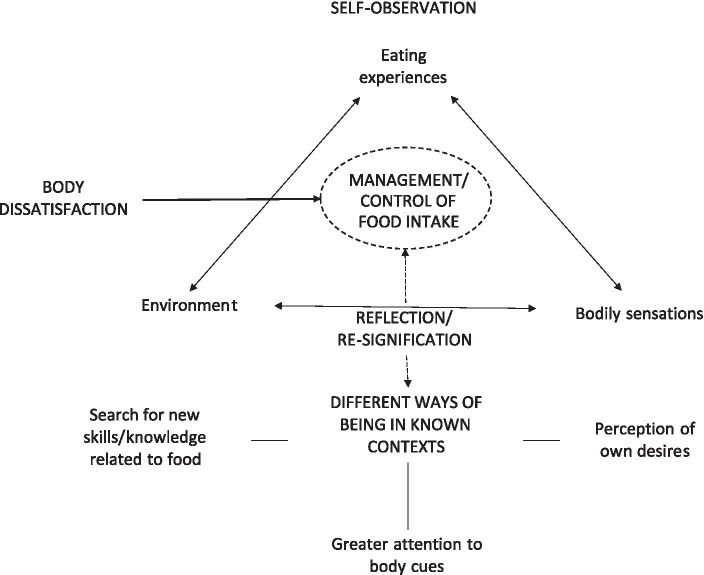
Interpretive map of the interviews.

The interpretive map (Figure 1) constructed from the interviews analysis is a visual support to help understand how the themes and sub-themes described and discussed below are related. Terms written in capital letters indicate the themes obtained from the analysis of the interviews.

#### 4.2.1 Food management or control

When talking about their relationship with food, the opposition made by many participants between the pleasure one has with food and what is considered adequate, here related to health and body aesthetic issues, is obvious. The idea of control arises from the search for balance between what one would like and what one should supposedly eat, which expands and connects to most of the topics covered.

Some reports are compatible with the eating behavior assessed by the cognitive restriction scale, i.e., the self-imposition of dietary rules to lose or maintain body weight. In this way, the foods consumed are constantly evaluated cognitively concerning quality and the ideal quantity to be consumed (Bryant et al., 2019).

#### 4.2.2 Body dissatisfaction

It is noted that the look and the attention directed to food appear in the participants’ lives from the dissatisfaction with the body, so this attention to the act of eating has the clear intention of exercising control over what is eaten and, thus, obtaining a body considered adequate. In this way, from the moment body dissatisfaction becomes present in the participants’ lives, eating ceases to be a spontaneous act, becoming the target of control and judgment.

In many reports, this idea is clear that eating only deserves attention because of the possible consequences associated with body weight and shape. The movement of attention to eating is related to negative sensations such as suffering, a sense of failure, and guilt.

This relationship between body dissatisfaction and attention to eating as a way of exercising control over food accompanied part of the participants for long periods, thus being a connection already well established at the beginning of the PESC workshops. In this way, attention exercises to body sensations in eating experiences were automatically associated with this intention of food control.

The appreciation of adequate weight and body shape considered acceptable and desired are contemporary characteristics directly related to food. Ideals of happiness, professional success, and social acceptance/inclusion are primarily linked to specific body standards, so the valuation of the thin body connected to the prejudice and negative judgment of the fat body spread by the media expands in a context in which body standards are taken as references in the search for a recognizable and globally valued identity. At the same time, the belief is strengthened that it is possible, through individual efforts – dietary restrictions, physical activity, consumption of nutritional supplements, and aesthetic procedures – to shape one’s body according to the desired shape. The idealization of a particular body type and the search to manipulate one’s body through dietary control result from the current social and cultural structure (Andrade and Bosi, 2003).

#### 4.2.3 Reflection and re-signification

The workshop activities constantly emphasized sensory aspects triggered by food-related environmental elements, followed by reflection on the usual developments in contemporary food experiences (Palazzo et al., 2021). The expansion of these reflections with connections to the participants’ eating habits and behavior was quite frequent in the reports obtained.

The intention of management and control over what is eaten at certain times is presented as a filter to the reflection on topics addressed in the workshops. The perception of the physiological sensations of hunger and satiety, or even the notion of flexibility as opposed to the rigidity of meal times or the amounts to be ingested at each moment, is interpreted by some participants as a path to greater control and reduction of the amount of food intake. In this way, the perception of body sensations and other sensations involved in eating experiences is validated as long as they serve the primary purpose of food control.

On the other hand, reflection on different everyday situations seems to promote the expansion of one’s own certainties and perspectives on food. Then, new possibilities and questions arise about what, until then, was conceived as sure.

The narrative expression promoted by various activities throughout the workshops and, ultimately, by the interviews means that perceptions can be externalized to become the object of reflection and re-signification so that they present a new form when re-internalized (Mollo et al., 2022). Within the perspective of the theory of consciousness used in this work, we can infer that this reflection and re-signification of perceptions is only possible in a context of conscious experiences and that this re-internalization of the reported contents occurs in an aware subject that is also modified and that from then on will have a new way of being in the world (Pereira Jr, 2013, 2014).

#### 4.2.4 Different ways of being in familiar environments

The perception of everyday sensations and processes that often went unnoticed led part of the participants to a process of self-knowledge and change of posture in the face of eating situations.

This reflective self-observation leads to new perceptions and considerations about food desires, the importance of body cues, and the need to search for new skills/knowledge related to food, which makes it possible to propose different ways of being in familiar environments.

The desire for food has a complex mechanism, triggered by sensory factors related to food and environmental or context-related factors (Shepherd, 2012), as well as the internal bio-psychic process. The desire for food is generally intensified by the mental simulation of the pleasure/satisfaction that can be felt when consuming a particular food (Papies et al., 2012; Shepherd, 2012). The elaborate-intrusive theory of desire proposes that food desire is triggered by often subtle environmental elements that generate intrusive thoughts - which suddenly pop into our minds (Kavanagh et al., 2005). These thoughts generate mental images that simulate with a wealth of sensory details the pleasure obtained by consuming specific foods and are directly related to greater food consumption of the concerned foods (May et al., 2012), which in part justifies the great success of food marketing strategies via social media (Folkvord and Hermans, 2020).

Making this process aware allows simple actions, such as turning off cell phone notifications, to change the conditions of environmental stimuli, which can result in changes in the intensity and frequency of food desires, which can also impact food consumption.

In many reports, greater attention is also described towards bodily sensations, in a movement to seek to perceive, discern and respond more to physiological desires and needs in different contexts.

The perception and appreciation of body cues are related to the balance of energy intake and, consequently, to maintaining body weight (Simmons and DeVille, 2017). This has stimulated the proposition and evaluation of interventions that seek to improve sensitivity (Herbert, 2020) and confidence in body cues (Tribole and Resch, 2012). The reports obtained show that the participants began to exercise the perception of bodily sensations in everyday situations outside the context of the intervention and also that this exercise was done because it made sense, because it had a meaning beyond the exercise itself, because it was something the participants saw as natural within their eating experiences.

Finally, reflections on eating situations also led to identifying the need and desire to seek new knowledge and skills related to food, especially involving the development of culinary skills.

Culinary skills are considered essential for acquiring and maintaining food autonomy and for establishing healthy eating practices as, when preparing their own food, individuals become more aware of what is actually consumed and less dependent on ready-to-eat food preparations available on the market (Diez-Garcia and de Castro, 2011), so searching for such skills is an essential step towards effective dietary changes.

### 4.3 Integration of questionnaires and interviews

Individual choices and, ultimately, eating behavior, result from a complex and dynamic interaction between biological and psychological aspects in response to environmental or physiological stimuli. Thus, given a context, sensory stimuli (such as the aroma or sight of food), or the physiological sensation of hunger, behaviors consistent with a repertoire of adaptive behaviors learned over time can be triggered (Dubé, 2010).

The great challenge for the brain is to regulate behavior to deal with different types of environmental and physiological stimuli and, at the same time, direct this behavior towards a specific objective, that is, to inhibit impulses, plan future actions, and enhance desirable and appropriate behaviors in a particular context (Dubé et al., 2008).

The tonic given to the control of food intake by the participants in the interviews can be seen as the result of the attempt to adapt practices and behaviors given the appreciation of the thin body, which, because it is associated with the idea of beauty, fullness, and acceptance, causes food and energy restrictions to be seen as something that will be reinforced in the long term, from the change in the body (Stroebe et al., 2013).

On the other hand, we have the high media, social, economic, and cultural appeal to the consumption of ultra-processed, hyper-palatable, and high-calorie foods. In addition to the cultural appreciation, low cost, easy access, and association of these foods to different events and social situations (Townshend and Lake, 2017), these foods exert a solid stimulus for the reward system, making their consumption result in highly pleasurable immediate sensations, which together configure a substantial appeal to excessive consumption (Weltens et al., 2014).

So, we have conflicting stimuli: on the one hand, the appeal to consume ultra-processed, hyper-palatable, and high-calorie foods and, on the other hand, the appreciation of thin bodies, which configures the action of two conflicting objectives. In an environment considered obesogenic, with broad availability and variety of food, the possibility of immediate food pleasure stands out, requiring more significant cognitive effort to maintain the goal of body weight control (Stroebe et al., 2013).

In this perspective, the expression of the intention to control is an attempt to regulate behavior in an environment that is hyperstimulating consumption. It should also be remembered that all participants in this research face difficulties in controlling body weight, which probably makes them seek to regulate food intake more emphatically, using the skills and resources available to them.

The evaluation using the TFEQ-R21 questionnaire did not indicate a change in the cognitive restriction scale after participating in the intervention, which reinforces the interpretation that the intention to control what one eats precedes participation in the workshops. Another result brought by the questionnaires that deserves attention is the increase in the IES-2 score, reflecting the increase in all its subscales, except the UPE (unconditional permission to eat), which is coherent within the perception that in an environment with excessive stimuli to food consumption, it would not be feasible to allow food consumption unconditionally.

Looking at the other IES-2 subscales, the increase in the scores for Eating for Physical Rather than Emotional Reasons, Reliance on Hunger and Satiety Cues, and Body-Food Choice Congruence confirm the greater attention and appreciation of body cues reported by the participants in group I-PESC, which is in line with the objectives of the PESC and, as described earlier, contributes to better dietary patterns (Smith and Hawks, 2006; Alberts et al., 2012; Warren et al., 2017; Richard et al., 2019; Schnepper et al., 2019).

The association between cognitive restriction and other dietary and anthropometric parameters is quite contradictory in the literature, with the hypothesis that restrictive behaviors may have positive or negative outcomes depending on other factors, such as flexibility or rigidity, in dealing with self-imposed rules (Stroebe et al., 2013; Bryant et al., 2019). Cognitive restriction can lead to greater difficulty in maintaining body weight due to factors such as lower sensitivity and responsiveness to satiety cues; loss of cognitive control in situations of emotional stress; and perception of diet violation when foods considered not allowed are consumed, which would lead to loss of motivation for the cognitive control of eating. This combination of factors means that, in a scenario where food stimuli surround us, the weight control goal is constantly challenged (Stroebe et al., 2013).

The decrease in the emotional eating scale score presented by the participants after the intervention points to a smaller loss of eating control in situations of negative emotions (Natacci and Ferreira Junior, 2011). There was an increase in the IES-2 score associated with this result, signaling a greater perception of bodily sensations, as opposed to vulnerability to environmental stimuli (Tylka and Kroon Van Diest, 2013). Changes in the relationship with food reported by the participants were also observed in the sense of reducing the occurrence of triggers for food desire and developing new food alternatives. These results allow us to infer that, after participating in the PESC, the intention to manage and control food became potentially more effective, less susceptible to oscillations imposed by the external environment, and more supported by internal resources.

The potential of this work resides in the evaluation through quantitative questionnaires and qualitative interviews of an innovative intervention, developed to promote consciousness of eating experiences through sensory and cognitive exercises. The evaluation of interventions of this nature is scarce in the literature so these results can motivate future investigations. The results discussed in this work point to the potential of PESC in the search for alternative approaches to promoting improvements in eating behavior.

The limitations of this work stem from the impossibility of randomizing the sample, given the conditions imposed by the coronavirus pandemic; the reduced sample size, which may have limited the results obtained through the questionnaires; the dedication time demanded from the participants, which resulted in the high rate of dropouts; the short intervention period, justified by the deadline for completing the research and the intention to reduce dropouts; and the lack of follow-up of the participants after the end of the intervention.

Reassessment after a certain period could provide information on the sustainability or otherwise of the results obtained over time. The challenges encountered in applying qualitative approaches in the health field must also be highlighted (Palazzo and Diez-Garcia, 2021). In this work, applying interviews in a clinical trial brought difficulties in constructing a listening and exchange space that could favor a more significant extension and depth of the reports obtained. Future studies may include a larger and diversified sample with different socioeconomic strata to better represent the general population in terms of access to food and willingness to make dietary changes.

## 5 Conclusion

This work evaluated an innovative intervention designed to promote consciousness of eating experiences through sensory and cognitive exercises. The proposition and evaluation of interventions of this nature are scarce in the literature, and frequently face theoretical and methodological challenges.

The evaluation of the PESC through the IES-2 and TFEQ–R21 questionnaires showed that after the intervention, the participants began to value body cues as a guide for food choices and reduced emotional eating in the face of emotional changes.

The analysis of the interviews indicated that the activities of the PESC promoted self-observation and reflection on different aspects of food that led to changes in the way of being in already familiar contexts, with identification and modification of triggers for food cravings, the intention of greater perception and appreciation of body cues and acquisition of new cooking skills/or food alternatives.

Together, these results demonstrate that stimulating the perception of bodily sensations related to eating situations led to changes in some aspects of eating behavior. PESC can improve food intake regulation based on a greater connection to internal bodily aspects and on a minor vulnerability to environmental stimuli for food consumption. The promotion of consciousness as a path to internal strengthening and reducing vulnerability to environmental stimuli is an innovative and promising aspect of the PESC that can positively contribute to promoting a better dietary pattern for individuals in environments with high food availability. The results of this work are auspicious and point to the potential of the PESC in the search for interventions to improve eating behavior that are not based on the traditional paradigm of diets.

## Data availability statement

The original contributions presented in the study are included in the article/supplementary material, further inquiries can be directed to the corresponding author.

## Ethics statement

The studies involving humans were approved by Research Ethics Committee of the Clinical Hospital of Ribeirão Preto (HCRP-USP). The studies were conducted in accordance with the local legislation and institutional requirements. The participants provided their written informed consent to participate in this study.

## Author contributions

CP: conceptualization, methodology, funding acquisition, investigation, visualization and writing - original draft preparation. BL: writing – review and editing. RD-G: conceptualization, funding acquisition, supervision and writing – review and editing. All authors read and approved the final version of the manuscript.

## References

[ref1] Alberts H. J. Thewissen R. Raes L. (2012). Dealing with problematic eating behaviour. The effects of a mindfulness-based intervention on eating behaviour, food cravings, dichotomous thinking and body image concern. Appetite 58, 847–851. doi: 10.1016/j.appet.2012.01.009, PMID: 22265753

[ref2] Anderson L. M. Reilly E. E. Schaumberg K. Dmochowski S. Anderson D. A. (2016). Contributions of mindful eating, intuitive eating, and restraint to BMI, disordered eating, and meal consumption in college students. Eat. Weight Disord. 21, 83–90. doi: 10.1007/s40519-015-0210-3, PMID: 26243300

[ref3] Andrade A. Bosi M. (2003). Media and subjectivity: impact on female feeding behavior. Rev. Nutr. 16, 117–125. doi: 10.1590/S1415-52732003000100012

[ref4] Braden A. Flatt S. W. Boutelle K. N. Strong D. Sherwood N. E. Rock C. L. (2016). Emotional eating is associated with weight loss success among adults enrolled in a weight loss program. J. Behav. Med. 39, 727–732. doi: 10.1007/s10865-016-9728-8 26931635 PMC5300743

[ref5] Braun V. Clarke V. (2006). “Using thematic analysis in psychology” in Qualitative research in psychology, 3, 77–101.

[ref6] Braun V. Clarke V. (2019). Reflecting on reflexive thematic analysis. Qual. Res. Sport Exerc. Health 11, 589–597. doi: 10.1080/2159676X.2019.1628806

[ref7] Bryant E. J. Rehman J. Pepper L. B. Walters E. R. (2019). Obesity and eating disturbance: the role of TFEQ restraint and disinhibition. Curr. Obes. Rep. 8, 363–372. doi: 10.1007/s13679-019-00365-x, PMID: 31701348 PMC6910890

[ref8] Chan A. Tetzlaff J. Altman D. (2013). SPIRIT 2013 statement: defining standard protocol items for clinical trials. Ann. Intern. Med. 158, 200–207. doi: 10.7326/0003-4819-158-3-201302050-00583, PMID: 23295957 PMC5114123

[ref9] Clifford D. Ozier A. Bundros J. Moore J. Kreiser A. Morris M. N. (2015). Impact of non-diet approaches on attitudes, behaviors and health outcomes: a systematic review. J. Nutr. Educ. Behavior 47, 143–155.e1. doi: 10.1016/j.jneb.2014.12.002, PMID: 25754299

[ref10] Cornelis M. C. Rimm E. B. Curhan G. C. Kraft P. Hunter D. J. Hu F. B.. (2014). Obesity susceptibility loci and uncontrolled eating, emotional eating and cognitive restraint behaviors in men and women. Obesity (Silver Spring) 22, E135–E141. doi: 10.1002/oby.20592, PMID: 23929626 PMC3858422

[ref11] Craig P. Dieppe P. Macintyre S. Michie S. Nazareth I. Petticrew M.. (2008). Developing and evaluating complex interventions: the new Medical Research Council guidance. BMJ 337:a1655. doi: 10.1136/bmj.a1655, PMID: 18824488 PMC2769032

[ref12] Creswell J. W. Clark V. L. P. (2013). Pesquisa de métodos mistos. 2nd Edn Porto Alegre: Penso Editora.

[ref13] da Silva W. R. Neves A. N. Ferreira L. Campos J. A. D. B. Swami V. (2018). A psychometric investigation of Brazilian Portuguese versions of the caregiver eating messages scale and intuitive eating Scale-2. Eat. Weight Disord. 25, 221–230. doi: 10.1007/s40519-018-0557-3, PMID: 30076529

[ref14] Diez-Garcia R. W. de Castro I. R. (2011). Culinary as an object of study and intervention in the field of food and nutrition. Cien. Saude Colet. 16, 91–98. doi: 10.1590/s1413-81232011000100013, PMID: 21180818

[ref15] Dubé L. (2010). “Introduction: on the brain-to society model of motivated choice and the whole-of-society approach to obesity prevention” in Obesity prevention. The hole of brain and society on individual behavior. ed. Bechara A. Dagher A. Drewnowski A. Lebel J. James P. Yada R. Y.. (Amsterdam: Academic Press).

[ref16] Dubé L. Bechara A. Böckenholt U. Ansari A. Dagher A. Smidts A. (2008). Towards a brain-to-society systems model of individual choice. Mark. Lett. 19, 323–336. doi: 10.1007/s11002-008-9057-y

[ref17] Ferreira S. C. Penaforte F. R. O. Cardoso A. S. R. da Silva M. V. T. Lima A. S. Correia M. I. T. D.. (2019). Eating behaviour patterns are associated with excessive weight gain after liver transplantation. J. Hum. Nutr. Diet. 32, 693–701. doi: 10.1111/jhn.12661, PMID: 31334582

[ref18] Folkvord F. Hermans R. C. (2020). Food Marketing in an Obesogenic Environment: A narrative overview of the potential of healthy food promotion to children and adults. Curr. Addict. Rep. 7, 431–436.

[ref19] Guivarch C. Charles M. A. Forhan A. Heude B. de Lauzon-Guillain B. (2022). Associations between maternal eating behaviors and feeding practices in toddlerhood. Appetite 174:106016. doi: 10.1016/j.appet.2022.106016, PMID: 35364113

[ref20] Herbert B. M. (2020). Interoception and its role for eating, obesity, and eating disorders: empirical findings and conceptual conclusions. Eur. J. Health Psychol. 27, 188–205. doi: 10.1027/2512-8442/a000062

[ref21] Herbert B. M. Blechert J. Hautzinger M. Matthias E. Herbert C. (2013). Intuitive eating is associated with interoceptive sensitivity. Appetite 70, 22–30. doi: 10.1016/j.appet.2013.06.082, PMID: 23811348

[ref22] Herbert B. M. Pollatos O. (2012). The body in the mind: on the relationship between interoception and embodiment. Top. Cogn. Sci. 4, 692–704. doi: 10.1111/j.1756-8765.2012.01189.x, PMID: 22389201

[ref23] Johnson R. B. Schoonenboom J. (2016). Adding qualitative and mixed methods research to health intervention studies: interacting with differences. Qual. Health Res. 26, 587–602. doi: 10.1177/1049732315617479 26657970

[ref24] Katcher J. Suminski R. Pacanowski C. (2021). An intuitive eating intervention improves dietary restraint, body appreciation, and intuitive eating in female undergraduates: a pilot study. Curr. Dev. Nutr. 5:977. doi: 10.1093/cdn/nzab051_021

[ref25] Kavanagh D. J. Andrade J. May J. (2005). Imaginary relish and exquisite torture: the elaborated intrusion theory of desire. Psychol. Rev. 112, 446–467. doi: 10.1037/0033-295X.112.2.446, PMID: 15783293

[ref26] Kristeller J. L. Wolever R. Q. (2011). Mindfulness-based eating awareness training for treating binge eating disorder: the conceptual foundation. Eat. Disord. 19, 49–61. doi: 10.1080/10640266.2011.533605, PMID: 21181579

[ref27] Kure Liu C. Joseph P. V. Feldman D. E. Kroll D. S. Burns J. A. Manza P.. (2019). Brain imaging of taste perception in obesity: a review. Curr. Nutr. Rep. 8, 108–119. doi: 10.1007/s13668-019-0269-y, PMID: 30945140 PMC6486899

[ref28] Kvale S. Brinkmann S. (2009). “Introduction to interview research” in Interviews: Learning from the craft of qualitative research interviewing (United States of America: Sage), 1–20.

[ref29] Lake A. A. (2018). Neighbourhood food environments: food choice, foodscapes and planning for health. Proc. Nutr. Soc. 77, 239–246. doi: 10.1017/S0029665118000022, PMID: 29493482

[ref30] Leghi B. E. Palazzo C. C. Magalhaes L. Diez-Garcia R. W. (2022). Using body-map storytelling for accessing insights in an educational intervention for food consciousness. Int J Qual Methods 21:160940692211169. doi: 10.1177/16094069221116972

[ref31] Lin Y. W. Lin C. Y. Strong C. Liu C. H. Hsieh Y. P. Lin Y. C.. (2021). Psychological correlates of eating behavior in overweight/obese adolescents in Taiwan: psychometric and correlation analysis of the three-factor eating questionnaire (TFEQ)-R21. Pediatr. Neonatol. 62, 41–48. doi: 10.1016/j.pedneo.2020.08.006, PMID: 32863168

[ref32] Lipschitz D. A. (1994). Screening for nutritional status in the elderly. Prim. Care 21, 55–67. doi: 10.1016/S0095-4543(21)00452-8 8197257

[ref33] Marshall C. Rossman G. B. (2016). “The how of the study. Building the research design” in Designing qualitative research. 6th ed. Marshall C. Rossman G. B. (Thousand Oaks: Sage), 99–138.

[ref34] May J. Andrade J. Kavanagh D. Hetherington M. (2012). Elaborated intrusion theory: a cognitive-emotional theory of food craving. Curr. Obes. Rep. 1, 114–121. doi: 10.1007/s13679-012-0010-2

[ref35] Mollo M. Iannaccone A. Savarese G. Pecoraro N. Fasano O. D’Elia D.. (2022). Reflective activity as a promoter of awareness processes in college students: a study. Front. Educ 7:7. doi: 10.3389/feduc.2022.835391

[ref36] Natacci L. C. Ferreira Junior M. (2011). The three factor eating questionnaire - R21: translation and administration to Brazilian women. Rev. Nutr. 24, 383–394. doi: 10.1590/S1415-52732011000300002

[ref37] O'Brien K. S. Latner J. D. Puhl R. M. Vartanian L. R. Giles C. Griva K.. (2016). The relationship between weight stigma and eating behavior is explained by weight bias internalization and psychological distress. Appetite 102, 70–76. doi: 10.1016/j.appet.2016.02.032, PMID: 26898319

[ref38] Palazzo C. Diez-Garcia R. (2021). Challenges on current practice of qualitative research: reflections and researcher positioning. Interface 25:210487. doi: 10.1590/interface.210487

[ref39] Palazzo C. C. Leghi B. E. Diez-Garcia R. W. (2022). Food consciousness intervention improves interoceptive sensitivity and expression of Exteroception in women. Nutrients 14:450. doi: 10.3390/nu14030450, PMID: 35276809 PMC8837977

[ref40] Palazzo C. C. Leghi B. E. Pereira-Júnior A. Diez-Garcia R. W. (2021). Educational intervention for food consciousness: a randomized study protocol. Nutr. Health 28, 123–129. doi: 10.1177/02601060211011801, PMID: 33913348

[ref41] Papies E. K. Barsalou L. W. Custers R. (2012). Mindful attention prevents mindless impulses. Soc. Psychol. Personal. Sci. 3, 291–299. doi: 10.1177/1948550611419031

[ref42] Pepino M. Y. Mennella J. A. (2007). Effects of cigarette smoking and family history of alcoholism on sweet taste perception and food cravings in women. Alcohol. Clin. Exp. Res. 31, 1891–1899. doi: 10.1111/j.1530-0277.2007.00519.x, PMID: 17949394 PMC2268904

[ref43] Pereira Jr A. (2013). “Triple-aspect monism: a framework for the science of human consciousness” in The Unity of mind, brain and world: Current perspectives on a science of consciousness. 1st ed Pereira Jr s. A. Lehmann D. (New york: Cambridge University Press)

[ref44] Pereira Jr A. (2014). Triple-aspect monism: physiological, mental unconscious and conscious aspects of brain activity. J. Integr. Neurosci. 13, 201–227. doi: 10.1142/S0219635214400068, PMID: 25012710

[ref45] Richard A. Meule A. Georgii C. Voderholzer U. Cuntz U. Wilhelm F. H.. (2019). Associations between interoceptive sensitivity, intuitive eating, and body mass index in patients with anorexia nervosa and normal-weight controls. Eur. Eat. Disord. Rev. 27, 571–577. doi: 10.1002/erv.2676, PMID: 30968474 PMC6767487

[ref46] Sandelowski M. (1996). Focus on qualitative methods: using qualitative methods in intervention studies. Res. Nurs. Health 19, 359–364. doi: 10.1002/(SICI)1098-240X(199608)19:4<359::AID-NUR9>3.0.CO;2-H 8773558

[ref47] Schnepper R. Richard A. Wilhelm F. H. Blechert J. (2019). A combined mindfulness–prolonged chewing intervention reduces body weight, food craving, and emotional eating. J. Consult. Clin. Psychol. 87, 106–111. doi: 10.1037/ccp0000361, PMID: 30570305

[ref48] Shepherd G. (2012). Neurogastronomy. New York: Columbia University Press.

[ref49] Simmons W. K. DeVille D. C. (2017). Interoceptive contributions to healthy eating and obesity. Curr. Opin. Psychol. 17, 106–112. doi: 10.1016/j.copsyc.2017.07.001, PMID: 28950955 PMC5657601

[ref50] Smith T. Hawks S. (2006). Intuitive eating, diet composition, and the meaning of food in healthy weight promotion. Am. J. Health Educ. 37, 130–136. doi: 10.1080/19325037.2006.10598892

[ref51] Stroebe W. van Koningsbruggen G. M. Papies E. K. Aarts H. (2013). Why most dieters fail but some succeed: a goal conflict model of eating behavior. Psychol. Rev. 120, 110–138. doi: 10.1037/a0030849 23230892

[ref52] Szakály Z. Kovács B. Szakály M. T. Nagy-Pető D. Gál T. Soós M. (2020). Examination of the eating behavior of the Hungarian population based on the TFEQ-R21 model. Nutrients 12:3514. doi: 10.3390/nu12113514, PMID: 33203100 PMC7696223

[ref53] Timmerman G. M. Brown A. (2012). The effect of a mindful restaurant eating intervention on weight management in women. J. Nutr. Educ. Behav. 44, 22–28. doi: 10.1016/j.jneb.2011.03.143, PMID: 22243980 PMC3259454

[ref54] Townshend T. Lake A. (2017). Obesogenic environments: current evidence of the built and food environments. Perspect. Public Health 137, 38–44. doi: 10.1177/1757913916679860, PMID: 28449616

[ref55] Tribole E. Resch E. (2012). Intuitive eating (St & M. S. Gdffin, Eds. 3rd ed., 1).

[ref56] Turjanski N. Lloyd G. (2005). Psychiatric side-effects of medications: recent developments. Adv. Psychiatr. Treat. 11, 58–70. doi: 10.1192/apt.11.1.58

[ref57] Tylka T. L. Kroon Van Diest A. M. (2013). The intuitive eating Scale-2: item refinement and psychometric evaluation with college women and men. J. Couns. Psychol. 60, 137–153. doi: 10.1037/a0030893, PMID: 23356469

[ref58] Victora C. G. Habicht J. P. Bryce J. (2004). Evidence-based public health: moving beyond randomized trials. Am. J. Public Health 94, 400–405. doi: 10.2105/ajph.94.3.400, PMID: 14998803 PMC1448265

[ref59] Wansink B. (2010). From mindless eating to mindlessly eating better. Physiol. Behav. 100, 454–463. doi: 10.1016/j.physbeh.2010.05.003, PMID: 20470810

[ref60] Warren J. M. Smith N. Ashwell M. (2017). A structured literature review on the role of mindfulness, mindful eating and intuitive eating in changing eating behaviours: effectiveness and associated potential mechanisms. Nutr. Res. Rev. 30, 272–283. doi: 10.1017/S0954422417000154, PMID: 28718396

[ref61] Weltens N. Zhao D. Van Oudenhove L. (2014). Where is the comfort in comfort foods? Mechanisms linking fat signaling, reward, and emotion. Neurogastroenterol. Motil. 26, 303–315. doi: 10.1111/nmo.12309, PMID: 24548257

[ref62] Zhang W. (2014). Mixed methods application in health intervention research: a multiple case study. Int. J. Mult. Res. Approaches 8, 24–35. doi: 10.5172/mra.2014.8.1.24

